# Identifying unmet needs in SSc-ILD by semi-qualitative in-depth interviews

**DOI:** 10.1093/rheumatology/keab154

**Published:** 2021-02-15

**Authors:** Anna-Maria Hoffmann-Vold, Elisabeth Bendstrup, Theodoros Dimitroulas, Roger Hesselstrand, Antonio Morais, Ritva Peltomaa, Vanessa Smith, Joep Welling, Madelon C Vonk, Wim A Wuyts

**Affiliations:** Department of Rheumatology, Oslo University Hospital, Oslo, Norway; Department of Respiratory Disease and Allergy, Centre for Rare Lung Diseases, Aarhus University Hospital, Aarhus, Denmark; Fourth Department of Internal Medicine, Hippokration Hospital, School of Medicine, Aristotle University of Thessaloniki, Thessaloniki, Greece; Section of Rheumatology, Department of Clinical Sciences, Skåne University Hospital, Lund, Sweden; Department of Pulmonology Centro Hospitalar, Universitário de São João, Faculdade de Medicina do Porto, Porto, Portugal; Department of Rheumatology, Helsinki University Hospital, Helsinki, Finland; Department of Internal Medicine, Ghent University; Department of Rheumatology, Ghent University Hospital; Unit for Molecular Immunology and Inflammation, VIB Inflammation Research Centre (IRC), Gent, Belgium; FESCA Federation of European Scleroderma Associations, NVLE, Utrecht; Department of Rheumatology, Radboud University Medical Centre, Nijmegen, The Netherlands; Department of Pulmonology, University Hospitals Leuven, Leuven, Belgium

**Keywords:** systemic sclerosis, interstitial lung disease, diagnosis, management, expert opinion, interview

## Abstract

**Objectives:**

Interstitial lung disease is frequent in SSc (SSc-ILD) and associates with significantly reduced quality of life. Here we aimed to analyse patient pathways, and experiences of patients and healthcare providers (HCPs) in order to identify unmet needs in the management of SSc-ILD patients.

**Methods:**

Semi-structured qualitative interviews conducted in eight European countries looked at HCP (*n* = 95) and patient perspectives (*n* = 47) using two sets of 70 research questions. Pre-diagnostic, diagnostic and post-diagnostic phases of the patient pathway were systematically explored.

**Results:**

(i) In the pre-diagnostic phase several gaps were identified by HCPs and patients in all participating countries: limited disease knowledge among primary care physicians and specialists, lack of accurate patient information, and delayed and/or inappropriate referral. (ii) The diagnostic phase is in most countries coordinated by rheumatologists, who are also the main point of care. Depending on the local health system, organization of multidisciplinary collaboration varies. HCPs issued lack of national guidelines, while patients stated difficulties obtaining disease-related information. (iii) In the post-diagnostic phase, HCPs and patients indicated lack of curative treatment, specialized nurses, and paramedical  and psychological support. Patients and caregivers additionally expressed the need for clear information on SSc-ILD.

**Conclusion:**

Lack of disease specific knowledge, gaps in national healthcare systems and insufficient information and support for patients and caregivers were identified as unmet needs to ensure timely diagnosis, provide better patient management and to improve quality of life in SSc-ILD patients.

**Rheumatology key messages**

- Semi-structured qualitative interviews conducted in eight European countries identified common unmet needs in SSc-interstitial lung disease management.
- Lack of knowledge and insufficient support for SSc patients and caregivers are the main unmet needs.
- SSc patients stated difficulties obtaining clear disease-specific information on SSc-interstitial lung disease.

## Introduction

Systemic sclerosis (SSc) is a rare CTD, characterized by fibrosis of the skin and internal organs [1–4]. Interstitial lung disease in SSc (SSc-ILD) frequently presents with widely heterogeneous severity of lung fibrosis, clinical course and functional impairment [5]. Regardless of severity, ILD has a major impact on the quality of life of the affected patients, and it is currently the leading cause of SSc-related mortality [5]. 

Particularly due to its heterogeneous presentation, management of SSc-ILD is challenging [6]. An international expert consensus has recently been developed on screening, diagnosis and monitoring of patients with SSc-ILD [7]. Treatment options for ILD in SSc patients are also increasingly available [8].

To secure implementation of these recommendations in daily clinical practice, it is important to identify remaining unmet needs in all phases of the patient journey [9–12]. These include pre-diagnostic, diagnostic and post-diagnostic phases, as experienced by healthcare providers (HCPs) and patients themselves [13]. It is well-known from other life-threatening diseases that actively involving patients in therapeutic decision-making is crucial for self-management and compliance [14]. Patient empowerment beyond treatment decisions has been an important focus worldwide over the past years. To develop well-functioning systems also for SSc, it is important to first identify their unmet needs.

This semi-qualitative research was conducted to gain in-depth insights in today’s management and care of SSc-ILD and to identify unmet needs and opportunities for improvement from the perspective of both HCPs and patients.

## Methods

### Study participants

The SClerOderma Pathway Exploration (SCOPE project) was performed in eight medium-sized Western European countries: Belgium, Denmark, Finland, Greece, Norway, Portugal, Sweden and The Netherlands. Attention was given to ensure balanced representation of the specialists involved in the management of SSc-ILD in the respective countries. Physicians from different disciplines (rheumatology, respiratory medicine, dermatology), nurses, patients and caregivers participated in the interviews. The physicians were selected based on their experience in SSc, i.e. at least 2 years of experience with SSc patients, currently treating SSc patients, recognized by their peers as experts in SSc or ILD, and their availability to participate in the interview. Patients and caregivers were recruited through the local patient associations and HCPs.

### Semi-qualitative interviews

**Fig. 1:**
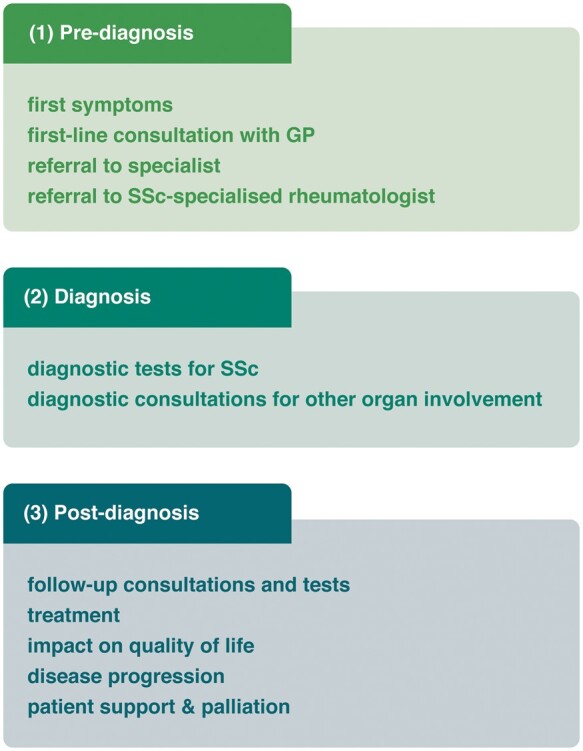
Overview of the consecutive phases of the patient journey in SSc and SSc-ILD GP: general practitioner; ILD: interstitial lung disease.

Semi-structured qualitative interviews were conducted, as described by Dicicco-Bloom *et al.* between October 2018 and February 2019 [15, 16]. The semi-structured design was chosen to give more control over the topics of the interview than in unstructured interviews, but in contrast to structured interviews or questionnaires that use closed questions, there is no fixed range of responses to each question. The researchers used a written, very specific and carefully worded questionnaire to guide the interviews. The four sets of around 70 research questions, adjusted to the interviewees’ profiles (specialist, patient, caregiver, patient organization) are available in the online material (Supplementary Data S1, available at *Rheumatology* online). Questions were asked during in-depth face-to-face or telephone conversations of about 1 h in a semi-directive approach, probing the same set of predetermined open-ended questions to all interviewees and adding questions that emerged during the interview. Interviews were conducted in the local language (Belgium, Netherlands, Portugal), in English (physicians in Denmark, Sweden, Norway, Finland, and Greece) or using a translator where needed (half of patients participating in Norway and Finland; Swedish and Danish patients were interviewed in English; no patient participated in Greece). All collected data were anonymized upon receipt. Three phases of the patient pathway were systematically explored (Fig. 1). This study is exempt from ethics review because it consists solely of qualitative in-depth interviews. All participants provided written informed consent before taking part in the study, and all study data were held according to European Union data protection laws.

## Results

### Participants

**Fig. 2:**
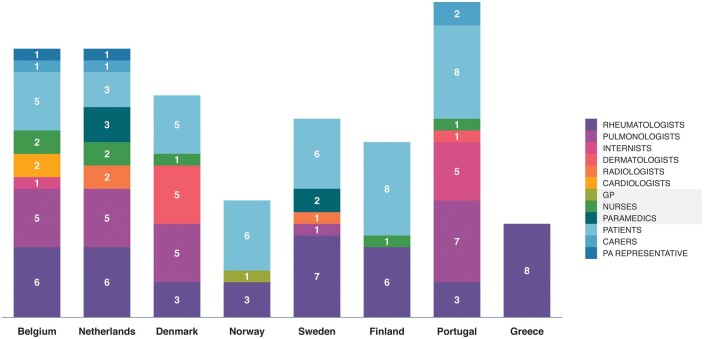
Number of semi-qualitative interviews performed per country GP: general practitioner; PA: patient association; PA representatives that are patient themselves (*n* = 4) are included in the patient groups.

A total of 142 participants were interviewed, including physicians (*n* = 83) from different disciplines, nurses (*n* = 7), paramedicals (two physiotherapists, two occupational therapists and one researcher), patients and caregivers (*n* = 47) (Fig. 2). Interviewees represented eight medium-sized countries, all with four to seven university or highly specialized centres in charge of SSc patients. Overall, 70 (74%) HCPs worked in university hospitals, 22 (22%) in non-academic centres, 2 privately and 1 in a health association. Of all HCPs, 39 (41%) were working in a centre with special interest in SSc; of these, 35 (90%) were linked to a university. Out of the 48 interviewed hospitals, 14 (29%) were considered locally as an ILD expert centre. All patients, except one, were treated in university or specialized centres.

Unmet needs in the patient pathway were very similar between countries (Table 1). Differences were related to specifics of the local healthcare system organization (Table 2).

**Table 1:** Unmet needs identified in the phases of the SSc patient pathway in all eight European countries

	Identified by HCPs and patients	Identified by HCPs	Identified by patients
Pre-diagnosis	Low awareness of first SSc symptoms among patients	Lack of clear referral structure	
	Limited knowledge of SSc among GPs		
	Low knowledge of SSc among specialists		
Diagnosis	Lack of knowledge regarding ILD	Absence of established care pathways	Patient confusion when receiving the diagnosis
	Difficulties for patients to remember information from the diagnostic consultation	Lack of specialized nurses	
Post-diagnosis	Lack of curative treatment	Lack of multidisciplinary collaboration among specialists	Difficulties in meeting other SSc patients for patients living in remote areas
	Lack of paramedical care		Difficulty in explaining the disease to others
	Lack of psychological support		
	Lack of clear and positive information		

GP: general practitioner; HCP: healthcare professional; ILD: interstitial lung disease.

**Table 2:** Overview of identified unmet needs in the respective countries

	Belgium	Denmark	Finland	Greece	Netherlands	Norway	Portugal	Sweden
Pre-diagnostic phase								
Lack of recognition/awareness of SSc-ILD expert centres	√				√		√	√
Competition between specialists and hospitals	√							
Gaps in primary healthcare						√		√
Diagnostic phase								
Lack of SSc guidelines	√		√			√	√	
Lack of SSc-specialized paramedicals	√	√	√			√	√	√
Lack of multidisciplinary meetings in peripheral hospitals	√					√	√	√
Pulmonologists’ lack of knowledge about SSc and unwillingness to be involved				√				
Post-diagnostic phase								
Lack of consultation coordination in hospitals	√							√
Main point of care’s lack of ILD awareness in peripheral hospitals	√						√	
Lack of palliative care			√			√		

ILD: interstitial lung disease.

### Unmet needs in the pre-diagnostic phase

Identifying SSc patients at the earliest disease stages is crucial in order to diagnose and intervene as early as possible. Time from first symptom to diagnosis is still 3 years on average [17]. Here, we assessed qualitatively patients and HCPs perceptions, and the issues raised were as follows.

#### Low awareness of first SSc symptoms among patients

Considering the first phase of the patient pathway, both HCPs and patients brought up that, due to insufficient knowledge, the first SSc symptoms usually do not trigger patients to seek advice from their general practitioner (GP). Patients are postponing consultation until symptoms worsen, additional symptoms appear or symptoms start having an impact on their activities of daily life. Even after SSc diagnosis, HCPs and patients found it difficult to attribute early symptoms to SSc because symptoms were mild in the beginning, were unspecific (e.g. RP, fatigue, gastrointestinal complaints) and because of the heterogeneous symptom pattern.

#### Low knowledge of SSc among GPs and (non-SSC) specialists

**Fig. 3:**
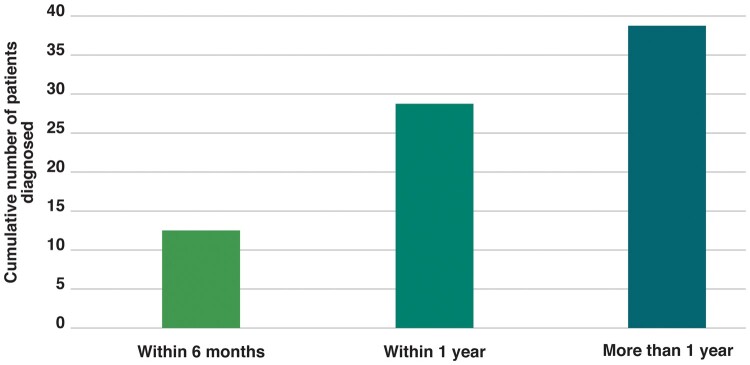
Time to diagnosis after first consultation in primary care

Knowledge of SSc was also considered insufficient in both primary and secondary care, leading to delayed referral and delayed diagnosis. However, large variations existed in the delay of referral to the SSc specialist from weeks to years, depending on the knowledge of the GP and the specialist who was consulted first. In more than half of patients participating in the interviews, diagnosis took more than a year (Fig. 3). The knowledge of SSc among specialists was often higher than among GPs, but many patients indicated that, also in secondary care, diagnosis took several months. Herein, the type of SSc and severity of symptoms were important determinants. Once the SSc specialist was consulted, diagnosis usually took only few weeks.

#### Lack of clear referral structure

HCPs indicated that referral structures are not clearly established, leading to delayed and/or inappropriate referrals and patients being managed by HCPs with limited knowledge of SSc.

Usually it takes several consultations before patients are referred to secondary care.

In most countries second-line SSc care is in the hands of rheumatologists, but also dermatologists or internists may be in charge. However, GPs refer their patients to several types of specialists, including specialists without specialization in SSc. In some countries, patients can visit specialists without referral.

### Unmet needs in the diagnostic phase

Patients and HCPs were concerned about diagnostic delays and consequently reduced quality of life. This concern was more pronounced in patients and patient associations representatives than in HCPs working at university centres.

In most participating countries rheumatologists are the main point of care for SSc and they coordinate the diagnostic work-up. Depending on the availability or structure of the SSc clinic, other specialists may also oversee the SSc patients, e.g. dermatologists or internists. Pulmonologists may also act as the main point of care, when ILD is the most prominent issue. Depending on the local healthcare system, the organization of multidisciplinary collaboration is varying. Pulmonologists are also involved in SSc-ILD patient care in all countries except for Greece. Their role is direct (Portugal, Finland, Denmark, Belgium) or indirect, supporting the rheumatologists (Netherlands, Sweden, Denmark, Norway, Belgium), unless transplantation is considered.

#### Lack of knowledge regarding ILD

Knowledge of SSc-ILD varied widely, depending on the rheumatologist’s SSc/ILD expertise, the collaboration between rheumatologists and pulmonologists, as well as the pulmonologist’s SSc/ILD expertise and involvement in SSc(-ILD). This limitation of knowledge was raised by both HCPs and patients.

Information on the diagnostic process was available in 40 patients: 19 (48%) reported that they also had been diagnosed with ILD. In 9 (47%) the diagnoses of SSc and ILD were made simultaneously; in the remaining 10 patients ILD was diagnosed at a later stage.

All interviews showed that SSc patients underwent lung function tests at the time of diagnosis, apart from some hospitals in Belgium where ILD screening was not always conducted. No structured information regarding high-resolution CT scan (HRCT) was collected.

#### Difficulties for patients to remember information provided in out-patient clinic

Patients indicated that they had difficulties in understanding and retaining the information that was shared during the diagnostic consultation. Patients and HCPs found that the confusion that resulted from receiving the unfavourable diagnosis was an important hurdle. Printed handouts or online materials were usually not available. Conversely, when patients had been informed about the risk of developing ILD at the time of SSc diagnosis, they were more receptive and able to capture disease-related information when ILD itself was diagnosed.

#### Absence of established care paths

All interviewed HCPs were diagnosing SSc and conducted comprehensive clinical assessment, nailfold video-capillaroscopy, lab tests (including SSc-specific autoantibodies), lung function tests, HRCT and ECG. National guidelines with established care paths on the diagnosis, treatment and follow-up of SSc patients have been developed only in the Netherlands and Denmark [18]. In Belgium, research and competence centres have been endorsed and are freely accessible online by the Royal Belgian Society. No other country had established care paths for SSc patients.

#### Insufficient supportive care

The interviewees also indicated as a shortcoming that, because of budgetary restrictions, many specialized centres do not have any assistance of specialized nurses to support patients during the diagnostic visit. Paramedical support, including physiotherapy and occupational therapy, was provided outside the hospital in most countries, close to the patients’ home (Belgium, Denmark, Portugal, Norway, Finland). Patients in Sweden could benefit from some sessions with specialized paramedicals in the hospital. Also, in the Netherlands patients may have sessions in the hospital.

### Unmet needs in the post-diagnostic phase

In the post-diagnostic phase life adjustments often are mandatory, new information is given and treatment initiated. The unmet needs identified in this phase were as follows.

#### Lack of curative treatment

According to the HCPs and patients, limited treatment options and the absence of a curative treatment are the main issues in this phase.

#### Lack of supportive care

Patients and HCPs agreed that there is a high unmet need for medical care, such as paramedical and psychological support. Physiotherapeutic sessions may be offered in the hospitals, but other paramedical professions are not mobilized, and patients and HCPs may not be aware of the benefits they can bring. HCPs also identified the fact that paramedicals are missing specific SSc training. Psychological support is available in all participating countries, but is rarely offered to patients. Many patients reported that they would have taken advantage of psychological counselling if it had been prescribed.

#### Lack of multidisciplinary collaboration for ILD

HCPs added lack of multidisciplinary collaboration among specialists as a barrier. Particularly in non-academic centres, insufficient collaboration between rheumatologists and pulmonologists may lead to suboptimal care of SSc-ILD patients.

#### Difficulties in communicating about the disease

SSc is a rare disease that is heterogeneous and unknown to most people. Patients found it difficult to explain their disease to others. Particularly patients living in remote areas experienced difficulties in meeting other SSc patients to share experiences with.

**Fig. 4:**
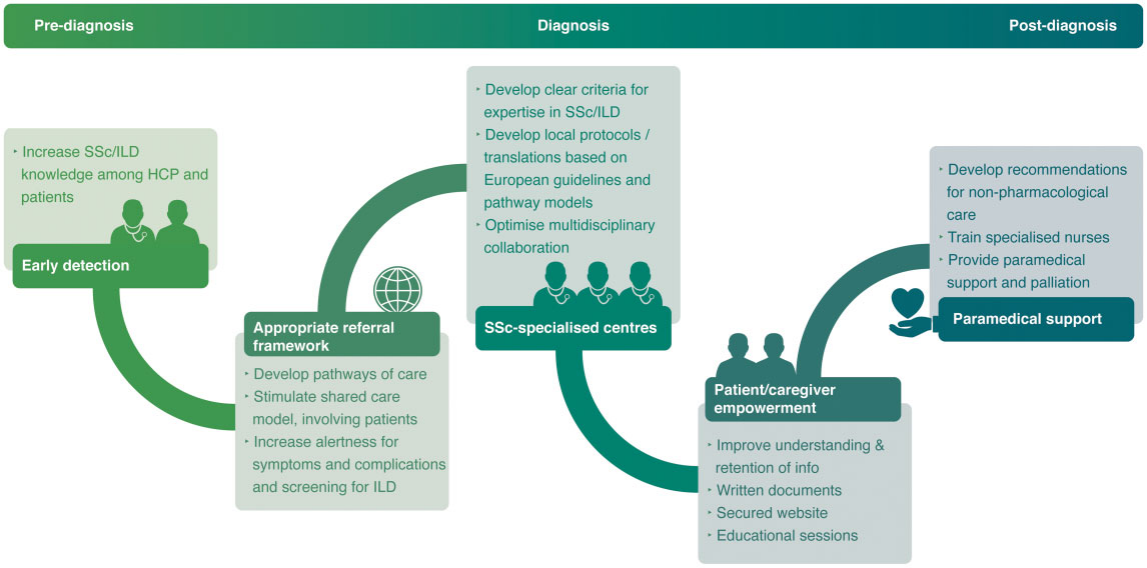
Practical suggestions to improve the patient journey in SSc and SSc-ILD HCP: healthcare professional; ILD: interstitial lung disease.

Based on the identified barriers, several recommendations can be made to improve the pathway of patients with SSc(-ILD) (Fig. 4).

## Discussion

Lately, there has been growing focus and awareness on SSc with increasing knowledge through real-world data collection, such as the EULAR Scleroderma Trials and Research Group (EUSTAR) network, randomized clinical trials and the development of SSc-ILD management consensus data [7, 19–21]. However, to date patients have not very frequently been involved and empowered in the development of recommendations and their own disease management. In this large semi-guided qualitative research study, including HCPs and patients, we identified several unmet needs in the current understanding and the management of patients living with SSc. We found 26 key issues in the pre-diagnostic, diagnostic and post-diagnostic phases, of which most are similar across countries. All are related to low disease knowledge among patients and peripheral physicians, lack of supportive care for patients or shortcomings in the national healthcare systems.

Long delays in diagnosis and treatment were identified, as well as lost early opportunities to optimize the quality of life in SSc, and even more in SSc-ILD, where approved treatment options have now become available [21]. Not only patients, but also first-line medical professionals had insufficient knowledge of the disease to identify patients at an early stage. These findings are aligned with recent work that was conducted among rheumatologists and dermatologists in larger European countries and the USA [22]. However, no patients were included in this study. Another large analysis of unmet needs in care has been carried out in patients living with idiopathic pulmonary fibrosis and HCPs in Europe [23]. The shortcomings they found in the patient pathway broadly overlap with our main findings in SSc-ILD: delays in diagnosis and in getting access to the ILD specialist and/or appropriate treatment requiring increased disease awareness.

Similar to the EULAR 2017 recommendations that—based on the complexity and heterogeneity of SSc and the limited amount of evidence—strive for the referral of patients with SSc to specialized centres with appropriate expertise in SSc management [6], the participants in our interviews raised the issue that referral structures are unclear and incompletely established. The SSc pathways of care in the Netherlands are a best-practice example of a clear referral framework [24]. Pathways of care are also helpful in creating disease awareness among physicians and may help in early detection of symptomatic patients and raise the alertness for complications, such as ILD. The shared care model was shown to be the preferred model of care and clear criteria for expertise in SSc are currently being developed [25].

The need for national guidelines is country dependent. The European guidelines are generally considered sufficient, but translations into local language make them accessible to every HCP. National guidelines have been developed for instance by the Danish National board on SSc. Some university hospitals have established their own protocol for the diagnosis of SSc, based on the European recommendations. Of note, state-of-the-art of existing clinical practice guidelines have been recently published by the European Reference Network (ERN) on Rare and Complex Connective Tissue and Musculoskeletal Diseases (ReCONNET) [18]. These networks aim to harmonize care throughout Europe and closely interact with national healthcare policies [26]. Alongside HCPs, patients also play a crucial role in these networks.

In the diagnostic phase, the lack of knowledge about ILD as a potential complication of SSc, or of SSc as an entity in treating patients with ILD, is particularly burdensome, as it may delay diagnosis and treatment. The recently published consensus statements show that all SSc patients should undergo screening for ILD, with HRCT and lung function testing [7]. Frequency of conducting follow-up HRCT should be guided by the risk for ILD, clinical symptoms and lung function [27]. Knowledge is important to implement this guidance in daily clinical practice. To increase the empowerment of patients, high quality information is crucial [28]. However, in this study both patients and HCPs indicated that patients have limited understanding of the disease, particularly at the time of diagnosis, when they are confused. It is well-known that during consultations, physicians often revert to medical terminology when the issue becomes complex, while patients use medical terminology without sufficient understanding of the terms [28, 29].

Patients and caregivers would also benefit from clear and reliable sources of information to help them communicate with HCPs, family and friends [30]. Written information and electronic information on secure websites may improve patients’ understanding and retention of the information provided during the consultation [31]. Educational sessions, such as the Learning and Mastery courses or Patient Information Days organized in Norwegian and Finnish hospitals are helpful to repeat the information that patients lost during the consultation, and to support patients and their caregivers in finding coping mechanisms.

Other patient solutions, such as telemedicine, could help patients living in remote areas. Listening to the patient’s voice is also an important aspect, as for instance in the Scleroderma Patient-centered Intervention Network (SPIN) project that teaches coping and disease-management skills to increase patient quality of life [32].

The Arthritis Research and Collaboration Hub (ARCH) study that questioned 650 SSc patients in the Netherlands concluded that three process indicators were of major importance: good patient–physician interaction, structural multidisciplinary collaboration and alignment of treatment with SSc guidelines [24]. They also found that patient education is an important point for improvement. The main source of information about SSc in this study was the physician. Patients also consulted the internet and websites of patient associations. Specialized nurses were important in the sharing of relevant information.

In our study the lack of specialized nurses and paramedical care was, however, an important unmet need in the patient pathway, mainly related to budgetary restrictions, which hamper the dissemination of adequate information. Non-pharmacological treatment is important for SSc-ILD patients, but here we also identified an unmet need for referral to rehabilitation and palliative care in SSc-ILD, as shown earlier in patients with idiopathic pulmonary fibrosis [33]. Patients with SSc-ILD would also benefit from other supportive care, e.g. occupational therapists and psychologists [33].

Our study is not without limitations, mainly based on its qualitative nature. The findings were subjective opinions of the interviewed patients and HCPs without validated translation of the questions. It should not, however, be confused with a validated, quantitative evaluation, which was not the objective of our study. Semi-qualitative in-depth interviewing is suitable to gain maximal insights but depends strongly on the interviewer’s competencies and participation of the interviewee. Therefore, maximal efforts were carried out to structure the interviews in a way that contributed to the objectivity of the results [16]. Another limitation was the selection of participants, which was based on SSc experience and willingness to participate in the interviews. Consequently, most were working in specialized centres and may have more positive views on the organization and results of patient management. This also explains why few GPs were involved, despite their important role in the early detection, which is an important limitation as they often are the first contacts for SSc patients. Patients able/willing to participate were more likely to be well informed about their disease and possibilities for care. Lastly, the under-representation of patients compared with medical professionals in some of the included countries may have had an impact on the perceived unmet needs in care from patients’ view point.

In conclusion, the lack of disease knowledge, gaps in national healthcare systems, and insufficient support for patients and caregivers were identified as the main unmet needs to overcome in order to improve quality of care and hopefully, preferably by a multidisciplinary approach, ensure timely diagnosis, provide better patient management and increase quality of life in patients living with SSc(-ILD).

## Supplementary Material

keab154_Supplementary_Data
