# Supplementary material for: Identifying unmet needs in SSc-ILD by semi-qualitative in-depth interviews
Source: Rheumatology (Oxford). 2021 Feb 15;60(12):5601–9. doi: 10.1093/rheumatology/keab154 (PMC8788003; doi:10.1093/rheumatology/keab154)
Supplement: keab154_Supplementary_Data [file keab154_supplementary_data.docx]

**Interview guides**

**THE SCOPE PROJECT**

*SClerOderma Pathway Exploration*

**i. hcps**

## General context

### For pulmonologists

1. How many SSc-ILD patients are diagnosed at your center every year?
2. How many ILD patients do you receive in your center every year? Among them, which proportion is affected by scleroderma?
3. How many SSc-ILD patients do you manage yourself?
4. Is there a typical scleroderma patient profile? *E.g.: average age, gender, others?*

### For other specialists (questions will be adapted to each specialty)

*The focus of this discussion is systemic sclerosis or scleroderma, in contrast to localized scleroderma.*

1. How many systemic sclerosis patients do you see at the moment? Is this number stable or is it increasing? How many new patients do you have per year?
2. Among them, do you know which proportion is affected by interstitial lung disease (ILD)^[[1]](#footnote-1)^?
3. How many SSc-ILD patients do you see every year?
4. Is there a typical scleroderma patient profile? *E.g.: average age, gender, others?* And is there a typical SSc-ILD patient profile?

### For GPs

1. Are you aware of scleroderma symptoms (*probe with scleroderma symptoms if the GP is not aware)*?
2. Do you have patients consulting for such symptoms?
   1. When they consult you for the first time, for how long had they those symptoms?
   2. What finally triggered their visit?
3. What are your decisions regarding these patients?
   1. Do you refer them to specialists? Which ones? Why?
   2. How long after their first consultation? After how many consultations in average?
4. To your opinion, how aware are GPs in general about scleroderma? What could be done to increase this awareness?

**For all specialists**

1. Has the number of systemic sclerosis patients at your center increased or decreased in recent years? What about SSc-ILD patients?
   1. To your opinion, what is the rationale behind this increase or decrease?
2. Regarding where your scleroderma patients normally get referred from:
   1. Are they usually in-patients? In that case, which hospital specialist is referring them to you?
   2. Are they usually out-patients? In that case, which type of HCP refers them to you? *E.g.: GPs, private specialists such as dermatologist, others?*
   3. Which proportion of your patients are in-patients?
   4. How do you collaborate with the referring practitioners?
   5. Are there any problem or good practices you are aware of? *[general question to give the interviewee the opportunity to express his/her opinion]*

## Pre-diagnosis: scleroderma

### First symptoms

1. What are the first symptoms of systemic sclerosis experienced by your patients? *E.g.: Raynaud phenomenon, cold and numb hands, swollen hands, others?*
2. How do patients experience these first symptoms? *E.g.: Are they stressed about the possible diagnosis? Do they mistake these symptoms with another disease?*
3. How severe are the first symptoms? How fast do they appear? *[Gradually vs. rapid worsening]*
4. Can different patient categories be distinguished already in the pre-diagnosis phase?

### First visit to a healthcare professional

1. At first, which doctor do patients with scleroderma symptoms usually visit? *E.g.: GP, dermatologist, others?*
2. What triggers the patient to visit a doctor for the first time?
3. How long after the first symptoms does the patient visit a doctor?
   1. Is this delay satisfying?
   2. What causes this delay?
   3. How could it be shortened?
4. What is the perception of the healthcare professionnals regarding the first symptoms of systemic sclerosis?
5. How aware are GPs about scleroderma in general?
6. How do patients experience their first visit to a doctor? *E.g.: concerned about the symptoms, stressed about the possible diagnosis, others?*

### First referral to a specialist (*if the patient saw a GP at first)*

1. At first, which specialists are patients referred to? *E.g.: dermatologist, pulmonologist, others?*
2. What triggers the GP to refer the patient to a specialist?
3. After how many consultations on average is the patient referred to a specialist? How long does it generally take? *[Days, weeks, months, years]*
   1. Is this delay satisfying?
   2. What causes this delay? *E.g. generality of the symptoms, others?*
   3. How could the referral to a specialist be faster?
4. What usually happens during the first consultation with a specialist? *E.g.: medical tests performed, key information provided to the patient, others?*
   1. Are patients referred to other specialists following this consultation? Which ones and why?
5. How does the patient experience the pre-diagnosis phase / what is his or her mindset at that stage?
6. How can the patient be better supported during this process?

## Diagnosis: scleroderma

1. Regarding the factors leading to a definitive scleroderma diagnosis:
   1. What are the key medical tests results?
   2. What are the key symptoms?
2. How easy is it to link these symptoms to scleroderma?
   1. How common are scleroderma symptoms? *E.g. general symptoms similar to other minor disease, specific symptoms but harder to detect, others?*
   2. Can they be mistaken with another disease? Which one?
3. What are the key tests conducted to diagnose scleroderma?
   1. How easy is it to conduct those tests?
   2. How long does it take to make all the tests?
   3. Who is conducting those tests?
   4. Where are those tests conducted? *[Primary, secondary or tertiary centers, when applicable]*
4. What type of specialist usually gives the final diagnosis of scleroderma?
5. Are there any other HCPs involved in the diagnosis of scleroderma?
   1. Which one? *[Other specialists, nurses]*
   2. What is their role?
   3. How do they communicate with each other?
   4. Is this level of communication or collaboration satisfying? What is lacking?
   5. How does this lack of communication impact the patient?
6. Regarding timing and delays:
7. How much time is there between the first symptoms and the final diagnosis? *[Days, weeks, months]*
8. How much time is there between the first visit to a healthcare professionals (usually GP) and the final diagnosis? *[Days, weeks, months]*
9. What is your opinion on this time window? Do you think this duration is satisfying? Why/Why not?
10. How could the diagnosis process be shortened?
11. What are the different treatments given to patients?
    1. What type of physician is provided the treatment? Only pharmacological or also non-pharmacological?
    2. Do all patients receive these treatments?
    3. ? What is your general impression about the adherence and compliance of patients to the treatments prescribed?
12. Among scleroderma patients, do you use any scientific sub-classification (limited scleroderma, diffuse or sine)?
    1. What about the distinction between limited scleroderma *[only parts of the skin]*, diffuse scleroderma *[all parts]* and sine scleroderma *[not the skin, rare]*?
13. What are the sub-classification criteria?
14. What is the proportion of patients who belong to one sub-classification or the other?
15. What are the differences in handling the different sub-classifications?
16. How do patient experience the scleroderma diagnosis phase? *E.g.: relieved to be diagnosed, fear of the future, others?*
17. What is the impact of the diagnosis on their daily life?
18. What are the supports available for patients diagnosed with scleroderma? *[Psychological and logistic support]*
19. What are the most crucial moments for the patient between the emergence of the first symptoms and the final diagnosis? *[Emotionally and medically?*

## Post-diagnosis: scleroderma progression

1. How does the centre take care of the patient after the diagnosis?
2. Who is the main caregiver or point of contact or the patients?
   1. Is the patient followed by a particular GP or specialist, or is the patient seeing different specialists for different symptoms?
   2. *[If specialists:]* How many different specialists does a scleroderma patient see on a regular basis? What type of specialists are they?
3. Is there any test conducted regularly to check how the disease is evolving? *E.g. DLCO, HRCT, FVC, mRSS, others?*
4. How often do patients have follow-up consultations?
   1. How do patients experience these multiple visits to specialists?
5. Is there multidisciplinary collaboration and communication?
   1. If yes, how do different specialists communicate with each other? *E.g. mail, letters, face-to-face meetings, others?*
   2. Is this level of communication or collaboration satisfying? What is lacking?
   3. What are the consequences of a potential miscommunication between these HCPs? *E.g.: potential negative drug interaction, necessity for the patient to re-explain his/her medical history at each new specialist involvement, others?*
   4. How could it be improved?
6. What are the main bottlenecks in patients follow-up?
7. How could this be improved?
8. After the diagnosis and all along disease’s evolution, what are the potential new symptoms which may develop over time? *[Probe respiratory symptoms]*
9. For each symptom *[mentioned in previous questions]:*
   1. How fast do they usually appear? *E.g.: progressive appearance, quick degradation and worsening of the symptoms, others?*
   2. Which treatments do they receive to cope with this symptom?
   3. Do all patients receive these treatments?
   4. What is you general impression about the adherence and compliance of patients to the treatments prescribed?
10. Are patients following several treatments for different symptoms at the same time?
    1. Is there a risk of drug interactions?
    2. How do you cope with this?
    3. Are there any studies conducted on drug interactions within systemic sclerosis?
11. Who can the patient contact when he/she experiences any issue during the treatment?
12. How?
13. Is it easy for patients to reach him/her? Why not?
14. Could that be improved? How?

## Pre-diagnosis: ILD

1. What are the differences between systemic sclerosis patients with ILD and ILD patients in terms of patient profile? *[Average age, gender]*

### First ILD symptoms

1. What are the first symptoms of ILD experienced by patients who are already diagnosed with scleroderma?
2. How long after the first scleroderma symptoms do patients usually develop ILD symptoms?
3. How do ILD symptoms differ from the symptoms experienced by people not affected by scleroderma?
4. How aware is the [your type of specialist] on the risk for patients with scleroderma to develop ILD?
5. What are the differences between the specialists? Are some specialists more aware of systemic sclerosis with ILD?
6. Which ones? Why?
7. How aware are SSc patients of being at risk to develop ILD?

### First referral to a pulmonologist

1. Who ultimately refers a scleroderma patient to a pulmonologist?
   1. What triggers this referral?
   2. How long after the symptoms onset does this referral take place?
2. How does the referral to a pulmonologist for SSc-ILD patient differ from a general ILD patient?
3. Does it happen to have patients firstly diagnosed with ILD and later on with scleroderma?
   1. How does this patient’s pathway differ from a primary SSc patient diagnosed later on with ILD?

## ILD Diagnosis

1. How does the diagnosis of systemic sclerosis patients with ILD differ from the one for ILD patients?
2. What are the tests performed?
   1. Who is conducting those tests?
3. Regarding timing:
   1. How much time is there between the first symptoms and the final diagnosis of ILD?
   2. How much time is there between the first visit to a pulmonologist and the final diagnosis?
   3. Is the diagnosis process faster or slower for patients already diagnosed with systemic sclerosis? Why?
   4. What is your opinion on this time window? Do you think this duration is satisfying? Why/Why not?
   5. Is there a need to optimize the referral process? If yes, why and how?
4. Are there several specialists involved in the diagnosis of ILD?
   1. Which specialist?
   2. If yes, how do they communicate?
5. How do patients usually receive their final diagnosis? *E.g.: during a consultation, by phone, by letter, others?*
   1. Who is involved?
   2. What are the aspects discussed during this conversation?
6. How do patients react to hearing their diagnosis? *E.g.: relieved to finally have a diagnosis, depressed and not asking too much questions, asking questions to know everything about the disease, denying that they have a chronic disease, fearing the future, others?*
   1. Do scleroderma patients react differently than other patients when hearing the ILD diagnosis?
   2. For scleroderma patients, what are the differences between receiving the ILD diagnosis and the scleroderma diagnosis?
7. What are the questions patients usually have at this point? *E.g.: questions on the disease onset, on the evolution of the disease on daily life, on the possibility to continue working, on possible treatments and side effects, on impact on their relatives, others?*
8. How do patients experience the ILD diagnosis test phase? *E.g.: are they confident in getting the right diagnosis? Are they insecure? Are they frightened about what disease they might have?*
   1. Do scleroderma patients experience this phase differently than ILD patients?
9. Who are the key persons supporting them during this stage and what are their roles? *E.g. relatives, psychologist, nurses, others?*

## Post-diagnosis: ILD

1. What are the differences between systemic sclerosis patients with ILD and SSc patients in terms of:
   1. Follow-up of the disease?
   2. Number and types of treatments followed?
   3. Impact of the disease on daily life?
   4. Frequency of visits to HCPs or treating center?
   5. Life expectancy?
2. How are patients followed-up after the ILD diagnosis?
3. How frequent and with whom?
4. What are the tests performed?
5. How do these consultations impact the patient’s life? *[Practical and emotional aspects]*
6. How do you coordinate with other involved HCPs once the patient is under treatment? Are there regular follow-ups between the HCPs?
7. What are the different treatments prescribed to systemic sclerosis patients with ILD?
   1. Do all patients receive these treatments?
   2. What is your general impression about the adherence and compliance of patients to the treatments prescribed?
   3. Do you see other bottlenecks linked to the pharmacological and non-pharmacological treatments of patients with SSc-ILD?
8. How do HCPs manage patient treatments when there are other diseases involved (other than ILD)?
   1. Is there a risk of drug interactions?
   2. How are potential drug interactions managed?
   3. Are there any studies conducted on this issue?
9. Are patients managed in a multidisciplinary way?
   1. If yes, how are different specialists communicating and exchanging about the patients? *[Channels, frequency]*
   2. Is this level of communication or collaboration satisfying? What is lacking?
   3. What are the consequences of a potential miscommunication between these HCPs? *E.g.: potential negative drug interaction, necessity for the patient to re-explain his/her medical history at each new specialist involvement, others?*
   4. Are there any bottlenecks in this multidisciplinary patient follow-up?
   5. What could be the potential solutions?

**ii. patients**

## General context

1. How old are you?
2. What is your family situation? *Children, partner?*
3. How long have you been diagnosed with scleroderma? And with ILD?
4. What does living with a chronic disease such as scleroderma with ILD mean to you?

## Pre-diagnosis: scleroderma

1. What were the first symptoms of scleroderma you experienced?
   1. When did they start?
   2. How severe were these first symptoms?
   3. How did you feel about these first symptoms? *Were you worried, or did it take time for you to pay attention to them?*
2. How long after the symptoms appeared did you visit a doctor? *[Days, weeks, months]*
3. What triggered you to visit to a doctor?
4. Which doctor did you visit at first? GP or specialist? Which specialist?
5. How did the symptoms impact your daily life during this period? *[Use a severity scale of 1-5, 1 being no impact, 5 being impact on basic daily activitie]*
   1. Physically
   2. Socially *[Social activities, relationship with family, friends]*
   3. Emotionally and psychologically
6. Did you receive any support during that time? If yes, what kind of support, and from whom?
7. What could have been done or done better to support you during this phase?
8. How did the symptoms evolve over time? Did new symptoms emerge with time?

## Diagnosis: scleroderma

1. How many specialists did you see before being diagnosed with scleroderma (at hospital or not)?)
   1. What type of specialist?
   2. Which specialist eventually diagnosed you?
2. Regarding the timing:
   1. How much time was there between the first symptoms of scleroderma and the final diagnosis?
   2. How much time was there between the first consultation with a specialist until the final scleroderma diagnosis?
3. How did you receive your diagnosis? *Letter, during a consultation, others?*
4. How did you react to the diagnosis? *Shocked, scared, others?*
5. What was the first thing you thought of?
6. What kind of emotion did you have? *E.g.: despair, anger, sadness, others?*
7. Had you already heard about scleroderma before, or was it the first time?
8. When first discussing about the diagnosis with the doctor or the nurse, how did you experience the conversation?
9. Was the diagnosis clear for you?
10. Which information did you receive during the consultation? What was the format?
11. Which specific questions did you have?
    1. Did you have time to ask all of them?
    2. Were you satisfied about the way they were answered?
    3. If not, where did you look for answers*? E.g.: patient forums, websites, others?*
12. Which information did you receive from the doctor about symptoms, treatment management, and evolution of the disease?
13. Were treatment options immediately discussed? If yes, what type of treatments: pharmacological or non-pharmacological?
14. What were the next steps mentioned by the HCP?
15. How did you feel after this consultation? *E.g.: relieved, depressed, others?*
16. Who did or didn’t you share the diagnosis with?
17. Why?
18. How important was it for you to share this diagnosis with people around you?
19. Regarding the reaction of people:
    1. How did your family react to hearing your diagnosis?
    2. How did friends, colleagues or others react? *E.g.: supportive, understanding, minimizing, others?*
20. Were you proposed any psychological support? Did you accept it, or did you look for support by yourself?
21. Was any other assistance offered to you? *E.g.: social worker, priest, others?*
22. Did you benefit from that assistance, was it useful and why?
23. What other type of support would have been helpful?
24. Were you put in contact with patient associations?
25. By whom?
26. Did you search for it yourself?
27. What was the role of the patient association? Was it useful for you, and why? What was lacking?
28. What was the biggest challenge for you during that period?
29. What could have been done or done better to support you during this phase?

## Post-diagnosis: scleroderma

1. Did you start any treatment once diagnosed with scleroderma?
   1. How many and what type of treatment?
   2. Did you follow several treatments for different symptoms?
   3. How easy was it for you to follow those treatments?
2. How many specialists do you see on a regular basis?
   1. What type of specialist?
   2. How frequently?
3. How did you feel before and after each follow-up consultation? Have you felt an evolution over the years? *E.g.: were you reluctant to go at first but now think that it is key?*
4. How do you feel about being taken care of by different HCPs?
5. Do you know if the specialists you regularly see communicate between each other? Or do you have to tell your story at each new specialist consultation?
6. Did new symptoms develop over time, since your diagnosis? Which ones?
7. Did you conduct any test to assess the disease evolution?
8. Who can you reach in-between the visits if you have questions or concerns? Could that be improved?
9. How has scleroderma impacted your life in terms of: *[Use scale of 1-5, 1 being limited impact, 5 being huge impact]*
   1. Physical discomfort
   2. Social life
   3. Physical activity
   4. Working life
   5. Psychologically
10. How has the disease impacted your family?
11. Who were the most important supports? *E.g. family, friends, patient association, others?*
12. What was the role of the patient association? *[If any]*
    1. What type of support was provided by the patient association?
    2. Was it helpful to better cope with the disease?

## Pre-diagnosis: ILD

### First symptoms

1. What were the first symptoms of ILD you experienced?
2. When did they start?
3. How did you feel about these symptoms at first? Were you worried, or did it take time for you to pay attention to them?
4. To whom did you talk about these ILD symptoms? *E.g.: family, friends, others?*
5. Were you aware that scleroderma could lead to ILD? Who explained that to you?

### First **referral to pulmonologist**

1. Were you first referred to another specialist than a pulmonologist? *E.g.: GP, radiologist, others? [If yes:]*
2. What happened during consultations with other specialists?
3. Who did they refer you to afterwards?
4. Who referred you to a pulmonologist eventually? Was this HCP involved in your scleroderma treatments already?
5. Regarding the timing:
   1. How much time was there between the first ILD symptoms (cough, dyspnea…) and the referral to the pulmonologist?
   2. How much time was there between your first consultation with a pulmonologist and the moment you were referred to the treating centre? *[Question to be adapted per country]*
6. What was your mindset before the first consultation with the pulmonologist?
7. Can you tell me a bit more about the consultation with a pulmonologist?
   1. Which tests did you perform?
   2. What were the outcomes of this consultation? *E.g.: first or definitive diagnosis? Referral decision?*
   3. Which information did you receive from the specialist?
8. Which specific questions did you have?
   1. Did you have time to ask all of them?
   2. Were you satisfied about the way they were answered?
   3. If not, where did you look for answers? *E.g.: patient forums, websites, others?*
9. What was your mindset after the consultation?
10. Did you have any challenges or concerns during that period? If yes, what were the major ones?
11. What did you do to face these challenges and to cope with your concerns?
12. Did you take any specific action? *E.g.: looking for information about the disease, searching for patient associations, others?*

## Diagnoses: ILD

1. When and in which structure did you receive your first diagnosis? And your final diagnosis?
2. Regarding the timing:
   1. How much time was there between the first ILD symptoms and the final diagnosis?
   2. How much time was there between the first consultations with a specialist until the final diagnosis?
3. How did you receive your diagnosis? *E.g. by letter, from a doctor, a nurse, others?*
   1. Was the way the diagnosis was communicated satisfying for you?
   2. Would you have preferred another way of communication?
4. How did you react to the diagnosis? *E.g.: relieved, depressed, others?*
5. What was the first thing you thought of?
6. What kind of emotion did you have? *E.g.: despair, anger, sadness, others?*
7. How did the fact that you are already affected by scleroderma impacted your mindset at that time?
8. Which specific questions did you have?
   1. Did you have time to ask all of them?
   2. Were you satisfied about the way they were answered?
9. Were treatment options immediately discussed?
10. What were the next steps mentioned by the HCP?
11. Who did or didn’t you share the diagnosis with?
12. Why?
13. How important was sharing this news to you?
14. Regarding the reactions of people around you:
    1. How did your family react to hearing the diagnosis?
    2. How did friends, colleagues or others react? *E.g.: understanding, minimizing, others?*
15. Were you put in to contact with patient associations?
16. By whom?
17. Did you search for it yourself?
18. Which patient associations?
19. What were the roles of the patient associations? Was it useful for you, and why? What was lacking?
20. Were you proposed any psychological support? Did you accept it, or did you look for support by yourself? if yes- how does this effect the current management of your disease (and then leave out Questions a and b).
21. If yes, how did psychological support helped you manage your disease?
22. What other type of support would have been helpful?
23. How does your disease impact your life? *[Use a scale of 1 to 5, 1 being limited impact, 5 being huge impact]*
    1. Daily life
    2. Emotionally and psychologically
    3. Socially
    4. Practically
24. How does it impact your family?
25. What was the biggest challenge for you when you received the diagnosis?
26. Who were the most important people for you during this period and why?
27. What could have been done or done better to support you during this phase?

## Post-diagnosis: ILD

1. On top of ILD, has scleroderma affected other organs? *[If yes:]*
   1. How do you manage the different treatments?
   2. What could be improved to better manage multiple-treatments?
2. How regularly do you come to the treating centre for follow-up visits?
3. What type of physicians do you see regularly?
   1. How many different physicians do you see?
   2. How do you feel about visiting different specialists? Do you know how the specialists communicate between each other? Do you have to tell your story at each new specialist consultation? )
4. Do you conduct any medical tests to the hospital on a regular basis to assess the evolution of the disease? Which ones and how often?
   1. Do you also conduct self-tests? Which ones and how regularly?
5. How do you feel before and after each follow-up visits? Have you felt an evolution over the years? *E.g.: were you reluctant to go at first but now think it is key?*
6. Who can you reach in-between the visits if you have questions or concerns? Could that be improved?
7. Who are the most important supports for you to cope with this disease?

**iii. carers**

## General context

1. How are you related to the person you are taking care of? *[Here referred as “your relative”, to be adapted]*
2. How long has [your relative] been diagnosed with systemic sclerosis? And with ILD?
3. What does it mean for you to live with someone having disease?

## Pre-diagnosis: systemic sclerosis

1. How did you feel about the symptoms [your relative] experienced at first? Were you worried?
2. Did [your relative] tell you about their symptoms since the beginning?
3. Did you take specific actions when you learned about the symptoms of [your relative]? *E.g.: looking for information about the symptoms, contacting people, others?*
4. Did you know what was systemic sclerosis before?
5. How did [your relative]’s symptoms impact you and your family at first?
6. What was the biggest challenge or what were your biggest concerns during that period?
7. What could have been done or done better to support [your relative], you and your family during this phase?

## Diagnosis: systemic sclerosis

1. Did you accompany [your relative] to the first consultation to a doctor?
2. How many consultations did [your relative] go to? How did he/she feel about this? *E.g.: worried, angry, others?*
   1. How did you feel during the consultation?
3. What was your mindset before the consultation? *E.g.: confident, worried, others?*
4. Did you hear about the diagnosis from the doctor or from [your relative]?
5. How did you react to hearing the diagnosis of [your relative]?
   1. What went through your mind when you heard it? *E.g.: scared, depressed, relieved to know what it is, others?*
6. During the consultations, did [your relative] receive enough information from the specialist?
   1. What about yourself?
   2. What questions did you have after hearing the diagnosis?
7. Was there any specific support offered for [your relative] at this stage? *[Psychological, educational, financial]*
   1. Was there any specific support for you?
8. With whom did you share [your relative]’s diagnosis? *E.g.: Family, friends, colleagues, others?*
   1. How did other family members react?
   2. How did friends, colleagues or others react? *E.g.: supportive, understanding, minimizing, others?*
   3. Did you have difficulties sharing the diagnosis? Why?
9. How could you and [your relative] have been better supported in this phase?

## Post-diagnosis: sclerosis

1. How is it to live with somebody suffering from systemic sclerosis? How did it change your daily life? *[Use scale 1-5, 1 being limited impact, 5 being high impact]*
   1. Practically
   2. Socially
   3. Psychologically
2. Did you go with [your relative] to all the medical follow-up visits?
3. How did you feel before and after each of these visits? Did you feel an evolution over the years? *E.g. you were reluctant to go at first but now you think it is important, others?*
4. What type of adjustment did you have to make in your life to cope with the disease of [your relative]?
5. What was the most difficult thing to cope with?
6. How did the disease evolve over time?
7. What were your biggest needs and concerns?
8. Did you receive support from a patient association? Was it beneficial for you?
9. What else would you have needed?

## Pre-diagnosis: ILD

1. Do you remember how long after the scleroderma diagnosis did [your relative] experience ILD symptoms?
2. Did [your relative] tell you about their symptoms right away?
3. How did you feel about the ILD symptoms [your relative] had at first? Were you worried?
4. Were you aware of the risk for systemic sclerosis patient to develop ILD?

## Diagnosis: ILD

1. How did you react to hearing the diagnosis of ILD of [your relative]?
   1. What went through your mind when you heard the diagnosis? *E.g.: scared, depressed, relieved to know what it is, etc.*
   2. Was it emotionally easier to receive the ILD diagnosis than the scleroderma diagnosis?
2. What questions did you have after hearing the diagnosis?
3. Was there any specific support offered to [your relative] at this stage? *[Psychological, educational, financial]*
   1. Was there any specific support for you?
4. What was the impact of ILD of [your relative] on your daily life?
   1. Practically
   2. Psychologically
   3. Socially
5. With whom did you share their diagnosis? *E.g. Family, friends, colleagues, others?*
   1. How did other family members react to hearing the diagnosis?
   2. How did friends, colleagues or others react? *E.g. supportive, understanding, minimizing, etc.*
   3. Did you have difficulties sharing the diagnosis?
6. How could you and [your relative] have been better supported in this phase?

## Post-diagnosis: ILD

1. How is it to live with somebody suffering from systemic sclerosis with ILD?
2. Do you go with [your relative] to all the medical follow-up visits?
3. How do you feel before and after each of these visits? Have you felt an evolution over the years? *E.g. were you reluctant to go at first but now you think it is important?*
4. What type of adjustment did you have to make in your life to cope with the disease of [your relative]?
5. What is the most difficult thing to cope with at the moment?
6. How did ILD evolve over time?
7. What are your biggest needs and concerns?
8. Do you receive support from patient associations? Is it beneficial for you?
9. Are you in contact with other patients suffering from the same disease?
10. What else would you have needed?

## Looking back at the entire process of diagnosis and disease management

1. Looking back at the whole pathway, which phase has impacted you the most emotionally and how/why?
2. What type of information sources were the most important for you?
   1. Why?
   2. At each phase of the pathway, what kind of information did you need the most?
   3. What was lacking the most?
3. Overall, what could be done or improved to support relatives like you from diagnosis onwards?
4. Overall, what could be done or improved to support patients from diagnosis onwards?

**iv. patient associations**

## General context

1. What is the role of your association?
2. What are the diseases targeted by your patient association?
3. Who is working within the organisation and/or involved in its activities?
4. What are your key activities? In particular, what are the activities conducted by *[name of patient association]* in the field of SSc/ILD?
   1. Are you conducting any activities to raise awareness?
   2. If yes, what type of activities and do you see an impact of those campaigns?
5. How many patient associations for *[SSc, ILD, SSc-ILD]* are there in *[country name]*?
   1. Do you collaborate with other patient associations for related disease? *[focus on scleroderma and pulmonary associations]*
   2. If yes, do you conduct joint activities?
   3. Do you think there are enough patient associations to support patients with *[SSc/ILD]* in *[country name]*?
6. How many *[SSc/ILD]* patients are member of your association?
   1. How many are suffering from scleroderma with ILD?
   2. Did the number of patients at your association increase or decrease during recent years?
   3. To your opinion, what is the rationale behind this increase or decrease?
7. Which activities/initiatives do you organize/coordinate for the following stakeholders and on what time basis?
   1. Patients
   2. Carers/families
   3. HCPs
   4. Pharmaceutical companies
8. Are following stakeholders involved in your organisation, and if yes, what are their roles?
   1. HCPs (GPs and specialists)
   2. Pharmaceutical companies
9. How and where can patients find out about your patient association? *E.g.*
   1. *Being referred by a GP, specialist, family, treating centers, etc.*
   2. *They actively look themselves for patient associations on the internet, social media*
10. Regarding the access to patient associations:
    1. How easy is it for patients to find and get in contact with a patient association?
    2. To what extend is contacting a patient association difficult for patients?
11. How many physical centers does the patient association have in *[country name]*?
    1. How easy is it for patients to get there physically?
    2. Do patients always visit the patient association in person or is the contact mainly done via phone and/or digital channels?
    3. Do you think this situation should be improved? If yes, how?
12. At which stage of their disease do patients usually contact/join your association? *E.g. immediately after the diagnosis, when starting medication, when the disease starts to worsen, etc.* Are you satisfied with this situation?
13. What kind of support are patients looking for when contacting your association? *E.g.*
    1. *Practical issues*
    2. *Emotional/psychological issues*
    3. *Physical issues*
    4. *Social issues*
    5. *Getting information on: the disease, treatment, side effects, impact of the disease on their lives, impact of the disease on their relatives*
    6. *To connect with other patients*
    7. *Managing daily tasks*
    8. *Other?*

## Stakeholders

1. What is the specific role of your patient association, compared to the role of other stakeholders (carers, HCPs)?
2. Regarding the communication and collaboration between different stakeholders:
   1. Does the patient association interact with other stakeholders?
   2. How do the different stakeholders of SSc-ILD patients (carers, HCPs, patient association, etc.) interact with each other?
   3. Could this multidisciplinary care be improved?
   4. If yes, how and what means are necessary?
3. Who is the main caregiver or point of contact of the patients?
   1. Is the patient followed up by a particular GP or specialist, or is the patient seeing different specialists for different symptoms?
   2. *[If specialists:]* How many different specialists does a patient see on a regular basis? What type of specialists are they?
   3. How do you think multidisciplinary care should be improve?
4. Who are the key persons supporting patients during the different stages and what are their roles? *E.g. relatives, psychologist, nurses, GP, specialist, patient association, etc.*
   1. Pre-diagnosis
   2. [SSc/ILD] diagnosis
   3. [SSc-ILD diagnosis]
   4. Post-diagnosis
5. What is the patient’s attitude towards each of the stakeholders? (trust/distrust)
6. GP
7. Specialists
8. Nurses
9. Carers
10. Relatives
11. Patient associations
12. Pharmaceutical companies

## Pre-diagnosis: systemic sclerosis

1. Is your association contacted by patients before they receive the SSc diagnosis?
2. Do you have any insight on how patients experience this pre-diagnosis phase?
3. What are the biggest challenges or concerns for patients during that period?
4. What could be done or done better (in general and by you as a patient association) to support patients and their families during this pre-diagnosis phase? *E.g. create awareness*
5. For many patients, a delay in the diagnosis is leading to unnecessary suffering, whereas earlier diagnosis would allow patients to start the treatment earlier. To your opinion, how could the pre-diagnosis phase be shortened? Is this mostly in hands of the patient, HCPs, carers, others?

## Diagnosis: systemic sclerosis

1. Who is involved in the diagnosis of scleroderma? *E.g. GPs, dermatologists, other specialists, etc.*
2. How do patients react to hearing their diagnosis? *E.g.*
3. *Relieved at first as they finally have a diagnosis*
4. *Depressed and not asking too much questions*
5. *Asking questions; wanting to know everything about the disease*
6. *Denying that they have a chronic disease*
7. *Fearing the future*
8. What questions does the patient have after receiving the diagnosis? What goes through their mind?
9. Questions on the disease onset?
10. Question on the progression of the disease?
11. Questions on whether he/she can continue working? (impact on daily life)
12. Questions on possible treatments and side effects?
13. Questions on impact on their relatives?
14. Is there specific support available for the patient at this stage (psychological, educational, financial, etc.)?
15. Who are the people involved in the support and what are their roles?
16. What type of support can your association provide to patients at that stage?
17. What are the biggest challenges for the patients and their relatives at this point?
18. How does the diagnosis of scleroderma impact the daily life of the patient *[use scale of 1-5, 1 being limited impact, 5 being huge impact]* and why?
19. Practically
20. Socially
21. Emotionally/psychological
22. Work
23. Physical discomfort
24. Other?
25. With whom do patients usually share their diagnosis? *E.g. Family, friends, colleagues, etc.*
    1. How do other family members react to hearing the diagnosis?
    2. How do friends, colleagues or others react? *E.g. supportive, understanding, minimizing, etc.*
    3. Do patients have difficulties sharing the diagnosis? If yes, why?
26. How can patients be better supported during the diagnosis phase?
27. How could the diagnosis phase be shortened? Is this mostly in the hands of the patient, HCPs, carers, others?

## E. Post-diagnosis: systemic sclerosis

1. What is the overall impact of scleroderma on the patients’ life? *[Use scale of 1-5, 1 being limited impact, 5 being huge impact]*
2. How does it change over time?
3. To what adjustments in their daily life does it lead?
4. What is the biggest challenge for patients to adapt to a life with scleroderma?
5. What are the most crucial moments for the patient in the post-diagnosis phase? Can you rank them according to their impact on the patient? *[Use scale of 1-5, 1 being limited impact, 5 being* *huge impact]* *E.g.*
6. *First worsening of the disease*
7. *Starting (new) medication*
8. *Experiencing (new) symptoms*
9. *Being diagnosed with ILD*
10. *Other?*
11. What is the overall impact of scleroderma on the relatives of the patient? *[Use scale of 1-5, 1 being limited impact, 5 being huge impact]*
    1. How does it change over time?
    2. To what adjustments in their daily life does it lead?
12. What are the support available for patients with SSc? Who is providing this support? Is this sufficient? *E.g.*
    1. *Programs/initiatives in hospitals/region, at national level, etc.*
    2. *Initiatives coming from your patient association*
    3. *Other organizations?*
13. According to you, what are the unmet needs of patients with SSc that should be better addressed? How? Who should be involved in this?

## F. Pre-diagnosis: ILD

1. How do patients generally feel about ILD symptoms at first? How worried are they?
2. Are scleroderma patients usually aware that they could develop ILD?
3. What are the biggest challenges or concerns for patients during that period?
4. What could be done or done better (in general and by you as a patient association) to support patients and their families during this pre-diagnosis phase of ILD? *E.g. create awareness*
5. For many patients, a delay in diagnosis leads to unnecessary suffering, whereas earlier diagnosis would allow the patients to start the treatment earlier. To your opinion, how could the pre-diagnosis phase be shortened? Is this mostly in the hands of the patient, HCPs, carers, others?

## G. Diagnosis: ILD

1. Who is involved in the diagnosis of ILD? *E.g. GPs, pulmonologists, other specialists, etc.*
2. How do patients react to hearing their diagnosis? *E.g.*
3. *Relieved at first as they finally have a diagnosis*
4. *Depressed and not asking too much questions*
5. *Asking questions; wanting to know everything about the disease*
6. *Denying that they have a chronic disease*
7. *Fearing the future*
8. What questions does the patient have after receiving the ILD diagnosis? What goes through their mind?
9. Questions on the disease onset?
10. Question on the progression of the disease?
11. Questions on whether he/she can continue working? (impact on daily life)
12. Questions on possible treatments and side effects?
13. Questions on impact on their relatives?
14. Is there specific support available for the patient at this stage (psychological, educational, financial, etc.)?
15. Who are the people involved in the support and what are their roles?
16. What type of support can your association provide to patients at that stage?
17. What are the biggest challenges for the patients and their relatives at this point?
18. How does the ILD diagnosis impact the daily life of the patient *[Use scale of 1-5, 1 being limited impact, 5 being huge impact]* and why?
19. Practically
20. Socially
21. Emotionally/psychological
22. Work
23. Physical discomfort
24. Other?
25. With whom do patients usually share their diagnosis? *E.g. Family, friends, colleagues, etc.*
26. How do other family members react to hearing the diagnosis?
27. How do friends, colleagues or others react? *E.g. supportive, understanding, minimizing, etc.*
28. Do patients have difficulties sharing the diagnosis? If yes, why?
29. How can patients be better supported in the diagnosis phase?
30. How could the diagnosis phase be shortened potentially? Is this mostly in hands of the patient, HCPs, carers, others?

## H. Post-diagnosis: ILD

1. What is the overall impact of ILD on the patients’ life? *[Use scale of 1-5, 1 being limited impact, 5 being huge impact]*
2. How does it change over time?
3. To what adjustments in their daily life does it lead *[on top of the adjustments made for scleroderma]*?
4. What is the biggest challenge for patients to adapt to a life with ILD?
5. What are the most crucial moments for the patient in the post-diagnosis phase? Can you rank them according to their impact on the patient? *[Use scale of 1-5, 1 being limited impact, 5 being* *huge impact]*

*E.g.*

1. *First worsening of the disease*
2. *Starting (new) medication*
3. *Experiencing (new) symptoms*
4. *Other?*
5. What is the overall impact of ILD on the relatives of the patients? *[Use scale of 1-5, 1 being limited impact, 5 being huge impact]*
   1. How does it change over time?
   2. To what adjustments in their daily life does it lead?
6. Is there any support available for patients with SSc-ILD? Who is providing this support? Is this sufficient? *E.g.*
   1. *Programs/initiatives in hospitals/region, at national level, etc.*
   2. *Initiatives coming from your patient association*
   3. *Other organisations?*
7. According to you, what are the unmet needs of patients with SSc-ILD that should be better addressed? How? Who should be involved in this?

## I. Looking back on the complete patient pathway

1. Looking back on the complete pathway, which phase impacts the patient the most emotionally and how/why?
2. Overall, what could be done or improved to support patients with SSc-ILD, from the first symptoms until the final diagnosis?
3. Overall, what could be done to improve the support to relatives of patients with SSc-ILD?

## J. Room for improvement

1. What is the level of awareness about *[SSc/ILD]* among the general public? What could be done to increase this awareness?
2. What is the level of awareness about *[SSc/ILD]* among HCPs (GPs and specialists)? What could be done to increase this awareness among the medical community?
3. To your opinion, what is the most important service a patient association can provide to a patient? *E.g.*
   1. *Psychological support*
   2. *Helping with practical issues*
   3. *Connect with other patients*
   4. *Provide them with the right information*
   5. *Inform their relatives*
   6. *Organisation of events*
   7. *Refer them to the right healthcare providers*
   8. *Other*
4. How could you, as a patient association, be able to help patients even better? *E.g.*
   1. *Get more financial means*
   2. *Get more people*
   3. *Organize more activities*
   4. *Get access to more tools, e.g. digital tools*
   5. *Other?*
5. In what way could pharmaceutical companies better support patients with *[SSc/ILD]*? E*.g.*
   1. *Create more awareness*
   2. *Support emotionally*
   3. *Inform patients in the right way*
   4. *Shorten time until first diagnosis*
   5. *Shorten time until treatment is started*
   6. *Other?*
6. Do you know HCPs in *[this country]* who are an expert in the field of SSc-ILD? Could you provide me with their contact details?
7. Do you know any other patient association linked to SSc-ILD? Could you provide me with their contact details?

1. *Explanation of ILD will be provided to non-pulmonologists* [↑](#footnote-ref-1)
